# Can Photobiomodulation improve healing of category II-III pressure ulcers in frail older adults? A quasi-experimental 24-month follow-up study

**DOI:** 10.1371/journal.pone.0359780

**Published:** 2026-10-28

**Authors:** Marianne Degerman, Bo Christer Bertilson, Micael Öhman, Laura Corneliusson, Marcus Schmitt-Egenolf, Åsa Audulv

**Affiliations:** 1 Department of Nursing, Umeå University, Umeå, Västerbotten, Sweden; 2 Department of Development, Skellefteå, Västerbotten, Sweden; 3 Division of Family Medicine and Primary Care, Department of Neurobiology, Care Sciences and Society, Karolinska Institutet, Stockholm, Sweden; 4 Division of Structural and Fire Engineering, Department of Civil, Environmental and Natural Resources Engineering, Luleå University of Technology, Norrbotten, Sweden; 5 Department of Public Health and Clinical Medicine, Dermatology, Umeå University, Västerbotten, Sweden; Massachusetts General Hospital, UNITED STATES OF AMERICA

## Abstract

**Background:**

Frail older adults are particularly susceptible to pressure ulcer development due to complex health conditions and functional decline. Pressure ulcers category II and III involve partial- and full-thickness skin loss, and current treatment options often yield suboptimal outcomes. The study evaluated the effects of adjunctive photobiomodulation compared with standard-of-care in frail older adults, with category II and III pressure ulcers. The objectives were to assess: 1) ulcer healing 2) healing time; 3) factors associated with healing, and 4) ulcer recurrence following photobiomodulation.

**Methods:**

Quasi-experimental study with two prospective intervention groups and matched registry-based control groups, conducted in accordance with TREND guidelines. Baseline differences analysed using Independent Samples T-test, Independent Samples Mann-Whitney U test or Pearson chi-square test. Times to healing evaluated using Kaplan-Meier analysis and log-rank tests. Age-stratified multivariable Cox proportional hazards models were applied to identify healing-associated factors. Ulcer recurrences were analysed descriptively.

**Participants and setting:**

Intervention groups comprised frail older adults (≥70 years) receiving municipal home healthcare: category II (n = 39) and category III (n = 37). Photobiomodulation was administered twice weekly, using GaAs laser (904 nm) and AlGaInP laser (635 nm). Control groups treated with standard-of-care were identified from the Swedish national registry RiksSår for ulcer treatment: category II (n = 117), and category III (n = 111).

**Results:**

The photobiomodulation groups demonstrated higher proportions of healed ulcers and significantly shorter healing times (*p* < 0.001). Median healing times were 33 and 70 days for the category II and III ulcers, respectively, compared with 153 and 195 days in the control groups. Ulcer recurrence within 24 months following photobiomodulation was 0% for category II and 6,7% for category III.

**Conclusions:**

Photobiomodulation may be an effective treatment for pressure ulcers in frail older adults, substantially accelerating healing, and reducing recurrence, with potential to lessen patient burden and improve outcomes.

**Trial registration:**

ClinicalTrials.gov NCT07520825

## Introduction

Frail older adults are particularly vulnerable to the development of hard-to-heal ulcers (HHU) [1,2]. The prevalence of HHU continues to increase in parallel with population ageing, and the healing outcomes remain suboptimal, resulting in substantial economic strain on healthcare systems globally [3,4]. Despite this burden, evidence supporting both established and emerging treatments in this population is limited, largely because frail older adults are frequently underrepresented or excluded from clinical trials [5]. Pressure ulcers (PU) are defined as a group of hard-to-heal ulcers that develop after sustained exposure to pressure and/or shear [6,7]. The risk of PU onset is elevated in individuals with immobility or declining physical function, where age, sex, malnutrition, and the presence of age-related comorbid conditions also have an important role [6,7]. Among individuals with neurodegenerative conditions, particularly advanced dementia, PU have been reported in close to 40% of patients in a terminal state [8]. Given the multifactorial origin and clinical complexity, PU necessitate a person-centred and holistic management strategy delivered by a multidisciplinary team. Such an approach should address the individual´s overall health status as well as aspects related to quality of life [1–3,7,9].

PUs are classified according to the international NPUAP/ EPUAP Pressure Ulcer Classification System, which distinguishes four main categories and two subcategories. Within this system, PU2 is defined as partial-thickness skin loss, while PU3 is characterised by full-thickness skin loss, in which subcutaneous fat may be visible although bone, tendon, or muscle remains unexposed [10].

Current guidelines for the management of PU encompass five key domains: evaluation and monitoring of ulcer healing, assessment and management of pain, ulcer cleansing and debridement, control of infection and biofilm formation, and selection of appropriate ulcer dressings [10].

Healing outcomes in PUs are influenced by ulcer severity, with more advanced categories associated with poorer healing prognosis, particularly in frail older adults [5]. In a home healthcare context, the healing prognosis of PU1 and PU2 was 24.4%, while category PU3 in general was in maintenance or deteriorating [11].

Recurrence rates following PU healing vary widely, a range of 5.4% up to 73.6% have been reported, with older adults and individuals affected by spinal cord injury or lower limb paralysis identified as having the greatest risk of recurrence [12].

Despite emerging evidence, Photobiomodulation (PBM) has not yet been incorporated into treatment recommendations for HHU [13]. PBM is a non-invasive, non-thermal light-based treatment that has not been associated with reported adverse events [14]. The biological effects of PBM are mediated through photochemical interactions within cells and tissues, influencing several stages of the ulcer healing process, including inflammation, proliferation, and tissue remodelling [13,15]. In addition, PBM has been associated with enhanced tissue regeneration and reductions in inflammation, pain and oedema [13–15].

The effectiveness of PBM in older adults has shown favourable results, supporting its use as a complementary and safe treatment for conditions such as cognitive disorders, Parkinsons disease, and ulcer aetiologies [16]. Investigations of PBM in ulcer treatment are promising. For example, showing enhanced tissue repair and a significant reduction in ulcer size in diabetic foot ulcers [17–22]. In studies on hard-to-heal venous leg ulcers [23] and arterial leg ulcers [24], frail older adults receiving PBM had significantly reduced healing time compared to control groups. Furthermore, a study investigating mixed categories of PUs treated with different PBM wavelengths reported positive effects of the use of 658 nm red wavelength on both tissue and serum outcomes [25]. Additionally, promising results, with clinical improvement and PU area reduction, were found in a scoping review [26]. Current evidence regarding PBM treatment for hard-to-heal ulcers suggests that the treatment would also be useful for PU; however, available literature remains limited. Many studies involve small sample sizes, inconsistently defined PU categories, and frequently exclude frail older adults. Furthermore, important determinants of ulcer healing, such as comorbid conditions, are often insufficiently addressed. Treatment protocols are commonly restricted to fixed durations or predefined numbers of sessions, and follow-up periods rarely extend beyond three months.

This study aims to investigate PBM treatment for PU categories two and three in frail older adults, namely to describe the difference in healing time between participants receiving PBM and control groups with standard-of-care, risk factors associated with healing, and the long-term durability of the treatment results. The research questions were:

- Do PU2 and PU3 treated with PBM have a higher probability of healing compared to standard-of-care treatment?
- Do PU2 and PU3 treated with PBM have a reduced healing time compared to matched control groups?
- Is there a difference in healing time between PU2 and PU3 treated with PBM?
- Does age, sex, comorbid conditions, physical function, ulcer duration, or ulcer area affect the healing time for PU2 or PU3 treated with PBM?
- What is the recurrence rate of PU within 24 months after healing with PBM treatment for PU2, respectively, PU3?

## Materials and methods

### Study design and ethics

This study is part of a prospective quasi-experimental project regarding PBM treatment for HHU in frail older adults receiving home healthcare; the protocol is registered in ClinicalTrials.gov with ID: NCT07520825 [27]. This study report results regarding treatments of PU2 and PU3, following the TREND guidelines [28]

The inclusion criteria were patients aged 70 years or older, with either PU2 or PU3 diagnosed by a physician. The study had four arms: PBM treatment groups for PU2 (PBM PU2) and PU3 (PBM PU3), as well as control groups PU2 (CG PU2) and PU3 (CG PU3). The division into ulcer type was important as PU2 and PU3 have different tissue depth and severity of tissue damage [10], and therefore different healing capabilities, including estimated healing outcome [5,11]. The two intervention groups received PBM treatment in addition to standard-of-care, and the control groups received standard-of-care.

The PBM groups were recruited from primary home healthcare patients in the municipality of Skellefteå receiving treatment for PU2 or PU3 between September 01, 2020 and May 30, 2023. Matched controls were identified in the National Swedish Quality Registry RiksSår for ulcer treatment [29]. The matching variables were PU category and ulcer duration at baseline; the CGs were 3:1 for the respective PBM group. Retrospective data were extracted from RiksSår on October 22, 2022. The enrolment of participants in the PU2 groups and the PU3 groups is shown in (Fig 1).

**Fig 1:**
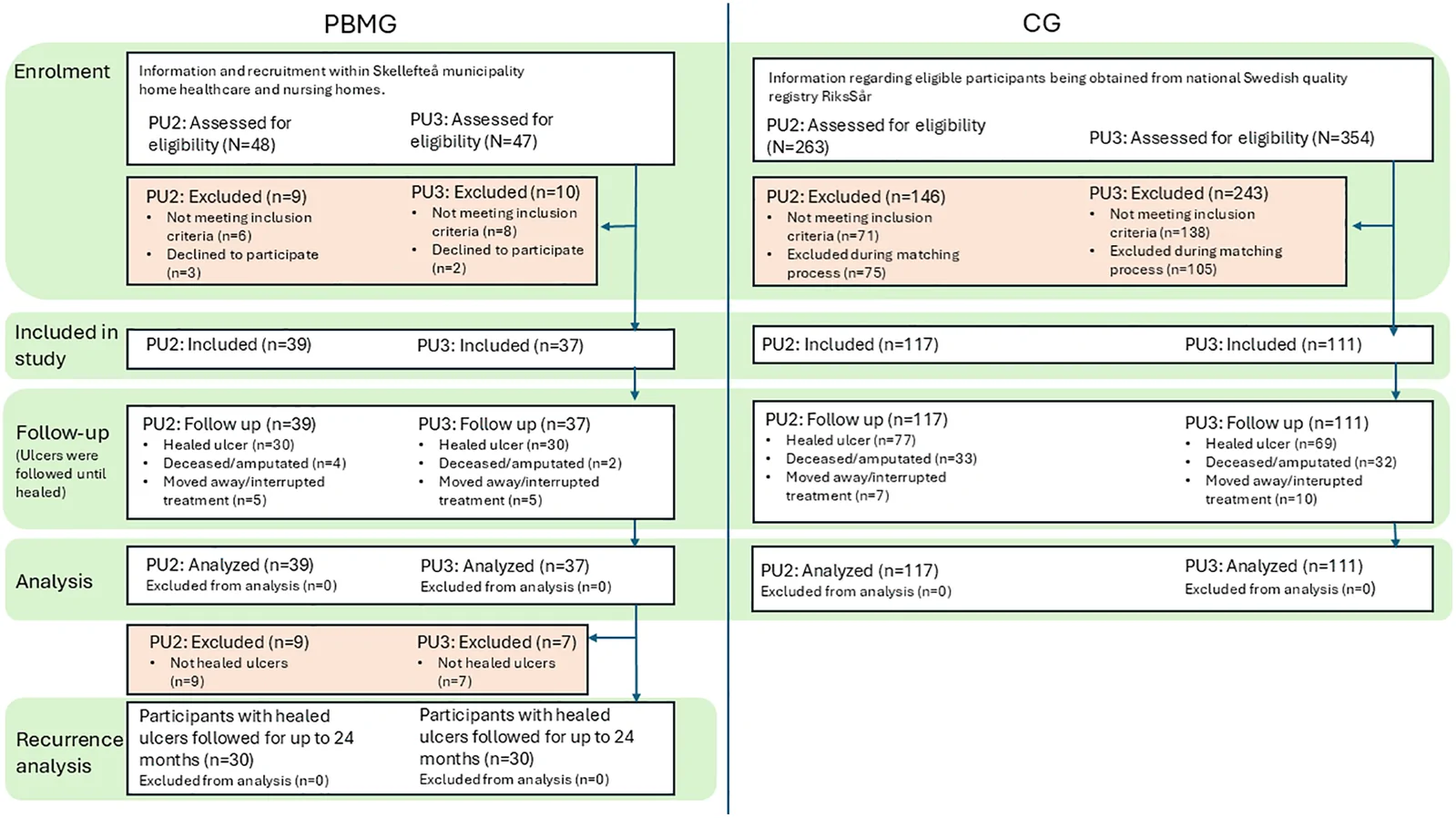
Enrolment of participants in the PBMG = photobiomodulation groups, and the CG = control groups, of PU2 = pressure ulcer category two, and PU3 = pressure ulcer category three.

Ethical approval for the study was obtained from the Swedish Ethical Review Authority in Lund, Sweden (Dnr. 2020−02194) with an amendment in 2024 (Dnr. 2024-00844-02). In the PBM groups, informed consent was obtained in writing from the participants, and when cognitive impairment was present, consent was additionally obtained from a legal guardian. The informed consent comprised participation in PBM treatment and, in the event of a healed ulcer, the collection of retrospective data from patient records up to 24 months after healing. A separate consent procedure was applied for inclusion in the RiksSår registry. All study procedures complied with the ethical principles outlined in the 2024 Declaration of Helsinki [30].

### Data material

Baseline data comprised demographic and clinical variables, including age, sex, number of comorbid conditions, physical function, ulcer duration, and ulcer area calculated as width x length in cm^2^. Physical function was assessed by a registered nurse and categorised as: wheelchair user/bedbound, walking with assistance, and walking. The outcome events recorded during the study period included healed ulcer, defined as: intact skin, amputation, deceased, and treatment interruption due to clinic transfer or treatment discontinuation. Time to healing was recorded in days. PU assessments were conducted two to three times per week until the occurrence of a treatment event.

To assess ulcer recurrence of PU2 or PU3 in the PBM groups, patient records were reviewed at 6-, 12-, 18-, and 24-months after healing. Recurrence was defined as the development of PU at the same anatomical site as the previously healed PU.

### Study procedure and selection criteria

Participants receiving PBM treatment were enrolled through a municipality-initiated project designed to examine PBM as an adjunct treatment for frail older adults with PU2 or PU3. PBM treatments were delivered by trained healthcare professionals in either the patients’ homes or in nursing home environments. Patient recruitment was carried out by nurses in cooperation with general practitioners. All eligible individuals who consented to participation were included. All participants in the PBM groups were assessed as frail according to the Clinical Frailty Scale [31]. Participants ranged 6–8 on a 0-to-9-point scale were higher number indicate severer frailty.

At study entry, all participants were confirmed to be receiving standard care for PU2 or PU3, in accordance with routine home healthcare practices, including individualised care plans and continuous risk monitoring using the Senior Alert register [32]. For ethical reasons, and to avoid exposing patients to unnecessary or ineffective treatment, the PBM intervention was evaluated after 6 weeks. If no evidence of ulcer healing or treatment response was observed at that time, PBM was discontinued. The CGs’ data were obtained from RiksSår, the Swedish national quality registry for ulcer treatment, which was established in 2009 [29]. At the time of data extraction, the registry covered approximately one-fifth of ulcer-treating facilities nationwide and contained over 20000 registered cases across multiple ulcer aetiologies. Data contributions originated from municipal primary home healthcare with 22%, primary healthcare centres and specialist hospital units with 43% and 35% respectively. Registry data was entered by the nurse responsible for each patient´s ulcer management [29].

### Treatment procedure

Each PU2 and PU3 were treated according to the physicians’ prescription with standard-of-care treatment based on the national recommendations for PU2 and PU3 treatment. Standard-of-care treatment includes offloading interventions, pressure redistribution, debridement, and ulcer dressings adapted to the PUs’ characteristics provided 2–3 times weekly [33]. Additional PBM treatment was administered during ulcer treatment, two times per week to the PBM groups, with intervals of 3–4 days.

### PBM treatment

Treatments were administered by a team of four healthcare professionals experienced in ulcer treatment and trained under supervision in PBM treatment, before study commencement. Total treatment duration varied between 20 and 40 minutes per session, depending on the PU size and the ulcer dressing complexity. Aspects such as exudate volume, ulcer area and ulcer location affected dressing complexity. The PBM treatment protocol and dosing parameters were developed based on recommendations from the World Association for Photobiomodulation Therapy (WALT) for the management of inflammation [34]. The parameters were further informed by prior PBM ulcer research [13,18–20,25] and tailored to the physiological profile of frail older adults. Treatment frequency was carefully considered to minimise exposure of PUs to ambient temperatures, which may temporarily delay ulcer healing [33], while ensuring that the therapeutic PBM dose was maintained. The treatment protocol included the use of both red and infrared wavelength PBM. Red wavelengths are suitable for treating superficial tissue [14] and are commonly used in PBM studies of ulcer treatment. While the 904 nm infrared wavelength was selected to enhance deeper tissue penetration and potentially influence lymphatic and circulatory function [14,35,36], which may be impaired in frail older adults.

The Infrared PBM was delivered using a handpiece containing 12 Gallium Arsenide (GaAs) laser diodes, each emitting light at a wavelength of 904 nm, with a continuous output power of 60 mW, a peak power of 25 W, and a pulse frequency of 700 Hz. The divergent laser beams had an aperture diameter of 5 mm when applied in a contact application. Red PBM was applied using an Aluminium Gallium Indium Phosphide (AlGalnP) laser with a wavelength of 635 nm, output power of 75 mW, pulse frequency of 250 Hz, and a divergent beam with a 9 mm aperture, when applied in a contact application.

The Infrared 904 nm PBM treatment was administered to the ulcer tissue and the lymphatic regions. Bilateral application was initiated at the shoulder/ neckline area for 120 seconds, delivering 7.2 J/cm^2^. Followed by treatment of the lymphatic area closest to the PU for an additional 120 seconds, delivering 7.2 J/ cm^2^. Intact skin surrounding the PU was then treated for 30 seconds at each location, proximal, distal and lateral to the PU, using a contact application technique, corresponding to a delivery of 1.8 J/cm^2^ per laser diode. The PU surface was subsequently treated using the projection application technique at a distance of 1 cm, with each position irradiated for 120 seconds, delivering 7.2 J per laser diode, until the entire ulcer surface was treated. Red 635 nm PBM was administered to both the ulcer margins and surface. Treatment of the ulcer edges was performed in 1 cm increments using contact application, with each position irradiated for 30 seconds, delivering 2.25 J/cm^2^. The PU surface was treated using the projection application technique at a distance of 1 cm, delivering 9 J per laser diode over 120 seconds per position, until complete PU surface coverage was achieved.

PBM treatment was delivered using Mid-Laser system (Spectro Analytic Irradia AB, Fagerstagatan 9, 163 53 Spånga, Sweden). Prior to study initiation, and subsequently at six-month intervals throughout the study period, as well as at study completion, the output from the handpieces was calibrated in accordance with the manufacturer’s guidelines [37].

### Statistical analysis

Data matching procedure three to one was performed for the CGs, where three PUs from RiksSår per PBM ulcer were matched on ulcer type and duration. The nearest match was extracted when an absolute match did not exist, and a random selection was applied when more ulcers than required existed. Statistical differences in baseline characteristics in the PBM group and the CG were calculated with an Independent-Samples t-test, an Independent-Samples Mann-Whitney U test, or a Pearson chi-square test for the two PU categories, separately. Baseline data were presented as descriptive statistics, percentages and p-values. Recurrence rates in the PBM groups were presented as descriptive statistics and percentages.

The time to ulcer healing was calculated using the Kaplan-Meier survival analysis method, and group differences were evaluated with the log-rank test. Deceased, interrupted treatment, and amputation were censored observations. To adjust for relevant clinical and demographic variables, hazard ratios (HR) and corresponding 95% confidence intervals (CI) were calculated applying an age-stratified multivariable Cox proportional hazards regression model. Three age groups, 70–79, 80–89, and ≥ 90 years, were included as a stratification variable, allowing for separate baseline hazard functions across age groups. The model included sex, physical function, ulcer duration, ulcer area, number of comorbid conditions, and treatment. These covariates included the baseline characteristics that differed significantly between the PBM and control groups and represented clinically relevant factors associated with pressure ulcer healing. A Fine and Gray competing-risk model analysis with subdistribution hazard ratios (SHR) and 95% CI was performed to account for the competing events of deceased and amputation, considering the variables ulcer area and number of comorbid conditions. A Time-varying Cox regression model with a time-dependent hazard ratio was calculated for the time points 14, 28, 42, 56, 70 and 100 days after baseline, respectively.

Alpha-level was set to 5%, 2-sided.

Statistical analysis was performed with the Statistical Package for the Social Sciences software program SPSS version 30.0 [38].

## Results

### Patient characteristics

The participants in the CGs had a more favourable health profile compared with the PBM groups. Regarding PU2, there was a statistical difference at baseline where the CG had smaller ulcer areas, fewer comorbid conditions and more male participants (*p* < 0,05). For PU3, the CG was significantly younger, had smaller ulcer areas and fewer comorbid conditions (*p* < 0.05). Neither PU category showed any significant differences in ulcer duration or physical function (Table 1).

**Table 1:** Participant characteristics.

Groups Number of ulcers	PBM PU2 39	CG PU2 117	P-value	PBM PU3 37	CG PU3 111	P-value
Age in years, mean/SD Age in years median/IQR	86.9/6.8 87.0/12	84.9/8.3 85.0/13	0.183 0.178	87.0/7.2 88.0/10.0	82.2/7.6 81.0/12	**<0.001** **<0.001**
Sex			**0.021**			0.502
Male, n/%	12/30.8	61/52.1		14/37.8	49/44.1	
Female n/%	27/69.2	56/47.9		23/62.2	62/55.9	
Ulcer area in cm^2^ mean/SD	23.0/49.7	5.9/16.8	**0.041**	5.5/5.3	3.6/4.6	**0.042**
Ulcer area in cm^2^ median/IQR	5.0/14.5	1.5/3.8	**<0.001**	4.0/5.2	1.9/3.8	**0.002**
Ulcer duration in weeks mean/SD	32.8/63.7	23.8/68.1	0.468	16.7/20.6	15.5/17.0	0.723
Ulcer duration in weeks, median/IQR	9.0/14	8.0/12	0.732	11.0/10	11.0/10.0	0.952
Number of conditions mean/SD	2.7/0.9	1.6/0.9	**<0.001**	2.7/1.0	1.7/0.8	**<0.001**
Number of conditions median/IQR	3.0/1.0	2.0/1.0	**<0.001**	3.0/1.0	2.0/1.0	**<0.001**
Physical function			0.929			0.805
Bedbound/Wheelchair user n/%	19/48.7	57/48.7		13/35.1	46/41.4	
Walking with assistance n/%	5/12.8	9/7.7		8/21.6	11/9.9	
Walking n/%	15/38.5	51/43.6		16/43.2	54/48.6	

Baseline data of: PBM PU2 group = Photobiomodulation pressure ulcer category two group; PU2 CG = Pressure ulcer category two control group; PBM PU3 = Photobiomodulation pressure ulcer category three group; PU3 CG = Pressure ulcer category three control group; SD = Standard deviation; IQR = Interquartile range; % = Percentage; n = number;

### Treatment outcomes

The two PBM groups had the highest percentage of healed PUs, with PU2 at 76.9% and PU3 at 81.1%, whereas the CG PU2 and CG PU3 had 65.8% and 62.2%, respectively. Censoring in the PBM groups was owing to deceased participant or interrupted treatment related to 1) Covid-19 pandemic: 5 PU2 and 4 PU3, 2) non-responding to PBM after six weeks, 1 PU3. Censoring in the CG PU2/ PU3 was owing to deceased participant, amputation, change of clinic or discontinuation of treatment. (Table 2).

**Table 2:** Treatment outcomes in the PU2 and PU3 groups.

Group	Healed n/%	Deceased n/%	Interrupted treatment n/%
PBM PU2	30/ 76.9	4/10.3	5/12.8
CG PU2	77/65.8	32/27.4	8/6.9
PBM PU3	30/81.1	2/5.4	5/13.5
CG PU3	69/62.2	32/28.8	10/9.0

PBM = Photobiomodulation group; PU2 = pressure ulcer category two; CG = control group; PU3 = pressure ulcer category three; n = number of ulcers, interrupted treatment due to a change of clinic, or discontinuation of treatment.

### Healing time

The PBM groups healed significantly faster than the respective control groups (p < 0.001). In the PU2 groups, the median healing time was 33 days in the PBM group, compared with 153 days in the CG. In the PU3 groups, the median healing time was 79 days in the PBM group, versus 195 days in the CG. (Fig 2) and (Table 3).

**Table 3:** Time to healing of PU2 and PU3 in days for each treatment group.

Group Healed	Mean SE_mean_	95% Cl_mean_	Median SE_median_	95% Cl_median_
PU(n)				
**PBM PU2 30**	59 13	34‒83	33 3	27‒39
**CG PU2 77**	207 20	168‒247	153 24	106‒200
**PBM PU3 30**	131 28	77‒186	79 19	42‒116
**CG PU3 69**	272 24	226‒319	195 21	154‒236

n = Number of healed; PU2 = pressure ulcer category two; PU3 = pressure ulcer category three; PBM = photobiomodulation group; CG = control group; SE = standard error; CI = confidence interval

**Fig 2:**
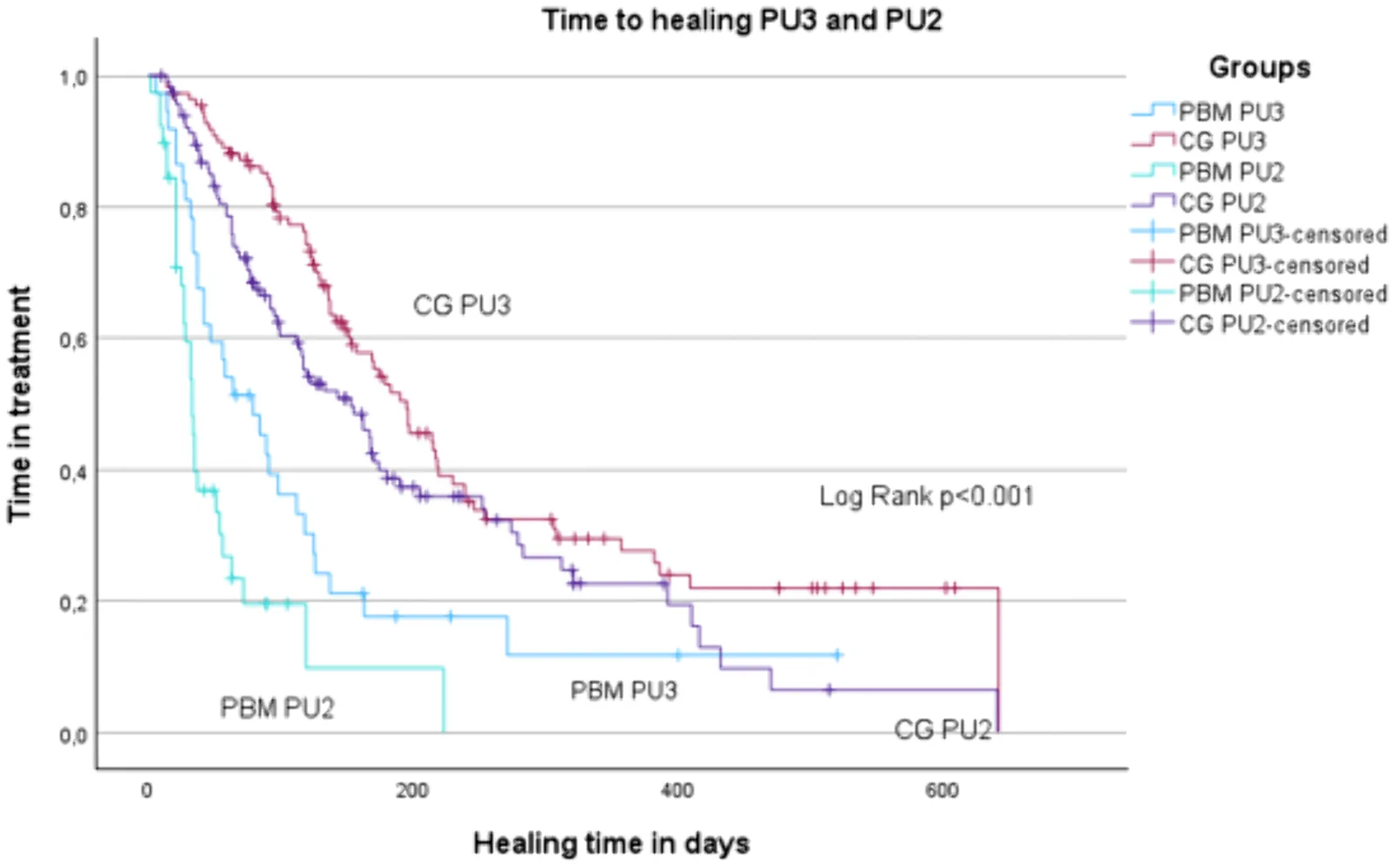
Kaplan-Meier curve of healing time of PU2 and PU3 = pressure ulcer category two and three; PBM group = Photobiomodulation group; CG = control group; Censored = deceased, moved away, or amputated.

Both the PBM PU2 and the PBM PU3 groups healed significantly faster than PUs in the two control groups. (Fig 2) and (Tables 3 and 4).

**Table 4:** Statistically significant differences in healing time between groups.

Group	Group	P value
PBM PU2	CG PU2	<0.001
PBM PU3	CG PU3	<0.001
PBM PU2	PBM PU3	0.008
PBM PU3	CG PU2	0.006
CG PU2	CG PU3	0.026

PBM = Photobiomodulation group; PU2 = pressure ulcer category two; CG = control group; PU3 = pressure ulcer category three, statistical significance calculated with the Log-rank test.

### Impact of different factors on healing time during treatment

For the PU2 groups and the PU3 groups, the age-stratified multivariable Cox proportional hazards regression models were fitted to evaluate factors associated with time to healing. The PU2 and the PU3 models demonstrated a significantly higher early healing hazard in the PBM groups compared with CGs, PU2 HR 95.512, 95% CI 27.293–334.240, *p* < 0.001, PU3 HR 195.828, CI 52.103–736.012, *p* < 0.001. A significant treatment-by-time interaction was observed in the models: PU2 HR 0.956 per day, 95% CI 0.936–0.977, *p* < 0.001 and PU3 HR 0.969 per day, CI 0.958–0.981, *p* < 0.001, indicating that the effect of PBM on time to ulcer healing changed over time. The time-specific hazard ratios showed that the hazard of healing associated with PBM was highest during the first weeks of treatment in both PU groups. At day 14, the HR was 59.501, 95% CI 21.767–162.653 for PU2 and 146.204, 95% CI 46.360–461.076 for PU3. At day 70, the corresponding HRs were 4.787 with 95% CI 2.096–10.936 for PU2 and 24.361, 95% CI 11.332–52.370 for PU3. Thus, although the relative treatment effect decreased over time, the estimated hazard of healing remained higher in the PBM groups throughout the treatment period. (Table 5).

**Table 5:** Time-varying hazard ratio of healing.

Time (days)	Hazard ratio PU2	95% Confidence interval PU2	Hazard ratio PU3	95% Confidence interval PU3
Interaction	0.956	0.936-0.977	0.969	0.958-0.981
14	59.501	21.767-162.653	146.204	46.360-461.076
28	31.690	14.101-71.219	93.410	33.452-260.837
42	16.878	8.427-33.802	59.680	23.814-149.563
56	8.989	4.459-18.118	38.130	16.678-87.386
70	4.787	2.096-10.936	24.361	11.332-52.370
100	–	–	9.328	4.444-19.578

Both models had a p-value of <0.001. PU2 and PU3 = pressure ulcer categories two and three.

In PU2, adjustment for sex, comorbid conditions, physical function, ulcer duration, and ulcer area did not materially alter the findings.

For the PU3 groups, the ulcer area was associated with a lower hazard of healing, HR 0.928, 95% CI 0.873–0.986, *p* = 0.017. Male sex was associated with a lower hazard of healing compared with female sex, HR 0.631, 95% CI 0.400–0.997, *p* = 0.048. Ulcer duration, number of comorbid conditions and physical function were not significantly associated with healing in the model. The results of the Fine and Gray competing-risk regression analysis of PU2 and PU3 were consistent with the age-stratified multivariable Cox proportional hazard regression models analysis. In PU3, the ulcer area was associated with SHR 0.934 and 95% CI 0.882–0.990, *p* = 0.021.

### Recurrence rate of PUs after healing with PBM treatment

In total, the recurrence rate was very low. In the PBM PU2, none of the PUs recurred, and after 24 months, 19 remained healed, and 11 participants had died with intact skin.

In the PBM PU3, two PUs out of 30 recurred (6.7%); both participants died shortly after the PUs had recurred. At the end of the follow-up time, 11 PUs remained healed, and 17 participants had died with intact skin. (Fig 3).

**Fig 3:**
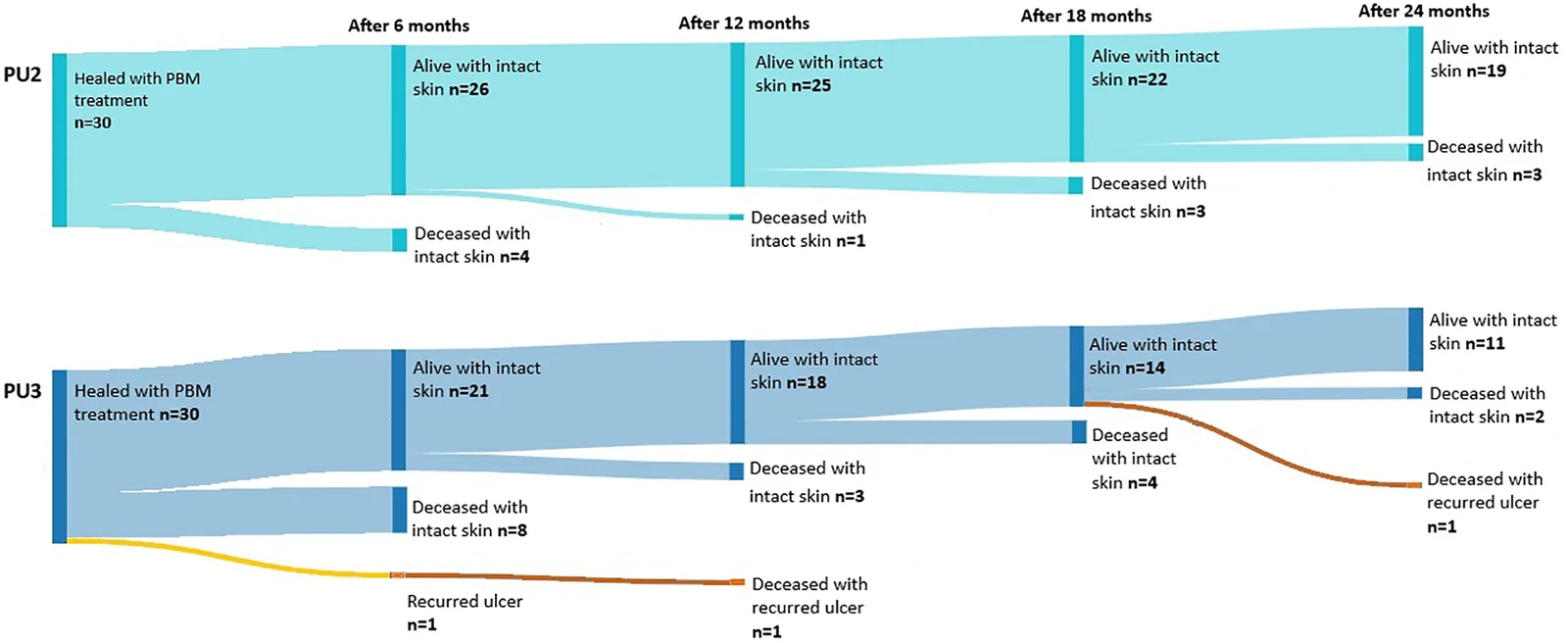
Recurrence rate of pressure ulcer category 2 and pressure ulcer category 3, over 24 months after healing with photobiomodulation.

## Discussion

In recent years, research has predominantly focused on the prevention of PU, which is a key aspect of patient safety and quality of care [6–8]. Nonetheless, up to 14% of frail older adults in nursing homes develop PU [39]. Consequently, more research is needed to investigate treatment options. The efficacy of current standard-of-care treatments for PU in older adults warrants further attention [5]. Prolonged PU duration has been shown to negatively affect patients’ quality of life and contribute to an increased risk of ulcer severity progression, infection, sepsis, and ultimately death [4,40]. In this study, the adjunct treatment with PBM showed that the proportion of healed PUs was greater in the two PBM groups compared to their respective CGs. Furthermore, the healing time was significantly shorter in the PBM groups (p < 0.001), with a median difference of 120 days in PU2 and 116 days in PU3 healing time. Surprisingly, the PU3s treated with PBM also healed significantly faster than the PU2s receiving standard-of-care, despite the substantial difference in PU severity. However, it should be noted that when comparing the two groups receiving PBM, the PU2s healed in about half the time compared to PU3, emphasising the importance of timely interventions to limit ulcer progression and prolonged treatment duration. The comparatively rapid healing observed in PU2 further underscores the value of early therapeutic initiation, suggesting that prompt implementation of PBM following ulcer onset may help prevent ulcer deterioration and lead to improved clinical outcomes.

Several covariates are known to influence PU healing time negatively. Patient-related factors include advanced age, the presence of comorbid conditions, reduced physical function, and sex [5,6,8], while ulcer-related factors include initial ulcer area and duration [1,2,6,8]. However, after adjusting for these variables, no significant associations with healing time were observed in the PU2 groups. Conversely, in the PU3 groups, a larger ulcer area contributed to prolonged healing time. Notably, the ulcer area was more advanced in the PBM group than in the CG, lending support to the reliability of the observed effect of the PBM treatment.

The healing durability over time was notable. There was no recurrence in the PU2s after healing with PBM, and the recurrence rate of PU3 was 6.7% over two years. This is substantially lower than recurrence rates previously reported for standard-of-care treatments with shorter follow-up periods, where rates of up to 74% have been observed [12]. Nevertheless, PU severity is a risk factor for recurrence, which is in line with prior evidence [12].

PBM may accelerate PU healing and reduce recurrence through combined local and systemic biological effects. At the cellular level, PBM enhances mitochondrial activity via cytochrome c oxidase stimulation, increasing adenosine triphosphate production and supporting cell migration, proliferation, and extracellular matrix synthesis [14,15], processes that are often impaired in patients with HHU. PBM also modulates reactive oxygen species at signalling levels, activating redox-sensitive pathways that promote angiogenesis and tissue repair [13,15]. In addition to local ulcer effects, the PBM treatment of lymphatic areas, included in this study, may contribute to the positive outcomes. The lymphatic system plays a critical role in fluid balance, immune regulation, and clearance of inflammatory mediators. PBM has been suggested to enhance lymphatic function and reduce interstitial oedema, thereby improving tissue oxygenation and creating a more favourable ulcer microenvironment [14,15]. Reduced oedema and more effective immune regulation may facilitate the timely resolution of inflammation, which is essential for progression to the proliferative phase of healing [41].

The lower recurrence rate observed in PBM-treated PUs may reflect not only faster ulcer closure but also improved tissue quality. PBM has been associated with enhanced collagen deposition, improved extracellular matrix organisation, and more balanced immune responses, potentially resulting in more resilient healed tissue and reduced susceptibility to ulcer re-formation [13].

It is possible that PBM has a preventive role in reducing PU risk in frail older adults by enhancing mitochondrial function and adenosine triphosphate availability, potentially improving tissue tolerance to ischemia and mechanical loading associated with immobility [14,15]. Additional effects on microcirculation, inflammation, and keratinocyte function may support skin integrity in pressure-prone areas [13,41]. Further research should investigate PBM as an adjunctive intervention for the preconditioning of at-risk, vulnerable skin in the prevention of PU. In this study, all participants had Fitzpatrick skin phototypes I-III [42]. Further research should include a broader range of skin phototypes to determine whether skin pigmentation influences PBM light transmission and treatment response in ulcer healing.

Previous results from this project have shown shorter healing time for PBM-treated hard-to-heal venous leg ulcers [23] and arterial leg ulcers [24] compared to standard-of-care. Taken together with existing research on PBM treatment for diabetic foot ulcers [17–22], the evidence suggests that PBM is effective for treating various aetiologies of HHU. PBM may be especially suitable for frail older adults given its non-invasive properties and the absence of reported adverse events. In addition, the accelerated healing and reduced recurrence observed in the PBM-treated HHUs have the potential to lessen patient burden by decreasing pain, immobility, and the risk of secondary complications such as infection or sepsis. Shorter healing times may also translate into decreased healthcare utilisation and associated costs, including reduced hospital stays, fewer nursing visits, and lower reliance on advanced ulcer care resources. Collectively, these findings highlight PBM not only as an effective therapeutic adjunct but also as a strategy with tangible benefits for both patients and healthcare systems.

Future research may explore the impact of PBM on patient-centred outcomes, including ulcer prevention, maintenance of independence, quality of life, and the health economic implications of PBM treatment.

### Limitations

This study employed a quasi-experimental design, which, unlike randomised controlled trials (RCTs), does not eliminate the risk of bias through random allocation [43]. To mitigate this limitation, matched CGs were used. Statistical differences between the PBM groups and the respective control groups at baseline were identified and reported. Notably, the PBM groups had a higher proportion of factors known to negatively influence healing, including more advanced age, a higher number of comorbid conditions and larger ulcer area [1,2,5,6,8]. Time-to-event and competing-risk analyses were conducted using the age-stratified multivariable Cox proportional hazard regression model, the Kaplan-Meier method and the Fine and Gray competing-risk regression, allowing adjustment for observed covariates and providing a structured comparison of healing outcomes over time [44–46]. Despite these measures, the possibility of residual confounding cannot be entirely excluded [44–46].

While RCTs are considered to provide the strongest evidence of internal validity, they may be difficult to implement in everyday clinical practice [43]. Quasi-experimental studies, although more vulnerable to certain biases, can therefore offer valuable insights into intervention effectiveness under clinical care conditions and in heterogeneous patient populations [45,47], such as patients receiving home healthcare.

## Conclusions

The findings suggest that PBM may be an effective adjunct treatment for PUs in frail older adults. PBM has the potential to accelerate PU healing, mitigate ulcer progression severity, lower recurrence rates, and alleviate patient burden. These results support consideration of PBM in clinical practice, while further research is warranted to evaluate the feasibility and cost-effectiveness of integrating PBM into routine care settings.

## Abbreviations

CG: Control group
CI: Confidence interval
AlGaInP: Aluminium Gallium Indium Phosphide
GaAs: Gallium arsenide
HHU: Hard-to-heal ulcer
HR: Hazard ratio
Hz: Hertz
IQR: Interquartile range
J: Joule
mW: milli Watt
nm: Nanometer
PBM: Photobiomodulation
PBM group: Photobiomodulation group
PU: Pressure ulcer
RCT: Randomised clinical trial
SD: Standard deviation
SHR: Subdistribution hazard ratio
SPSS: Statistical Package for the Social Sciences
W: Watt
